# Advanced Imaging-Guided Intravenous Thrombolysis Beyond 4.5 Hours: A Single-Center Experience in Mexico

**DOI:** 10.7759/cureus.117686

**Published:** 2026-10-09

**Authors:** Ricardo E Jiménez Gatica, Erika E Gonzalez Sansores, Daniel E Velasco Hernández, Ilse C Blancas Hernández, Alejandra Calderón Vallejo, Beatriz Méndez González

**Affiliations:** 1 Endovascular Therapy, Specialty Hospital “Dr. Bernardo Sepúlveda Gutiérrez,” Siglo XXI National Medical Center, Mexican Institute of Social Security, Ciudad de México, MEX; 2 Neurology, Specialty Hospital “Dr. Bernardo Sepúlveda Gutiérrez,” Siglo XXI National Medical Center, Mexican Institute of Social Security, Ciudad de México, MEX; 3 Endovascular Neurology, Specialty Hospital “Dr. Bernardo Sepúlveda Gutiérrez,” Siglo XXI National Medical Center, Mexican Institute of Social Security, Ciudad de México, MEX; 4 Neurological Endovascular Therapy, Specialty Hospital “Dr. Bernardo Sepúlveda Gutiérrez,” Siglo XXI National Medical Center, Mexican Institute of Social Security, Ciudad de México, MEX

**Keywords:** acute cerebral infarction, cerebro-vascular accident (stroke), extended time window, intravenous thrombolysis, stroke

## Abstract

Introduction

Intravenous thrombolysis is the standard treatment for acute ischemic stroke within the first 4.5 hours; however, many patients present with unknown symptom onset or delayed presentation. Advanced neuroimaging allows identification of salvageable brain tissue and extension of the therapeutic window up to nine hours, and recent evidence suggests that, in carefully selected patients, reperfusion may be beneficial up to 24 hours after stroke onset.

Objective

The objective of this study is to report the institutional experience with advanced imaging-guided intravenous thrombolysis in the extended therapeutic window, including its frequency of use and workflow times.

Methods

We conducted a retrospective, descriptive study including consecutive patients with acute ischemic stroke who received intravenous thrombolysis between March 2022 and May 2025. Patients treated beyond 4.5 hours after symptom onset or with unknown onset time were identified and analyzed. Eligibility for thrombolysis was determined using computed tomography (CT) perfusion or magnetic resonance imaging (MRI) mismatch criteria. Descriptive analyses were performed using IBM SPSS Statistics for Windows, Version 27 (Released 2019; IBM Corp., Armonk, NY, USA).

Results

Fourteen patients (17.7%) out of a total of 79 were identified. Female sex predominated (79%), with a mean age of 67 ± 17 years and a median National Institutes of Health Stroke Scale (NIHSS) score of 9 points. The most frequently affected territory was the anterior circulation (79%), primarily the middle cerebral artery. Onset was unknown in 36% of cases. Median time from symptom onset was 327 minutes; door-to-imaging time was 30 minutes, and door-to-needle time was 74 minutes. No in-hospital mortality was recorded.

Conclusions

Advanced imaging-guided thrombolysis is feasible in tertiary care centers in Mexico, thereby expanding access to reperfusion therapy for patients with delayed presentation.

## Introduction

Ischemic stroke is considered the second leading cause of death and the leading cause of non-traumatic disability worldwide [1,2], accounting for 143 million disability-adjusted life years [3,4].

Pharmacological thrombolysis is a reperfusion therapy whose main objectives are to restore blood flow and limit brain tissue damage [5], and it has proven highly effective when administered within the first 4.5 hours of symptom onset, constituting the standard of care in the acute phase [6,7]. However, a substantial proportion of patients are excluded from this intervention due to delayed arrival at emergency services or difficulty in precisely establishing the time of symptom onset [8].

Advances in neuroimaging techniques, particularly diffusion- and fluid-attenuated inversion recovery (FLAIR)-weighted magnetic resonance imaging (MRI), as well as computed tomography (CT) perfusion studies, have allowed more precise characterization of the ischemic penumbra and distinction of potentially salvageable tissue from the established infarct core [9,10].

In this context, clinical trials such as WAKE-UP [11], EXTEND-IA [12], and ECASS-IV [13] have provided solid evidence supporting the safety and efficacy of intravenous thrombolysis in an extended window between 4.5 and 9 hours, provided rigorous selection criteria are applied [14,15]. More recently, the possibility of intravenous thrombolysis up to 24 hours has been explored in clinical scenarios where transfer to a referral center is not feasible; the TRACE-III trial used tenecteplase up to 24 hours after symptom onset, reporting better functional outcomes compared with standard medical treatment [16].

Extended-window thrombolysis thus represents an opportunity to broaden therapeutic options and optimize acute stroke care [17-19].

The present study aims to report the frequency of extended-window or unknown-onset thrombolysis and to describe the clinical characteristics and care times of patients selected by advanced imaging studies at a tertiary care center in Mexico.

Despite growing international evidence supporting imaging-guided thrombolysis in the extended window, data from middle-income countries such as Mexico remain limited. Differences in healthcare systems, in-hospital care times, availability of advanced neuroimaging, and organization of stroke teams may significantly influence the implementation of these protocols. It is therefore relevant to document the experience of referral centers in order to better understand the frequency of use of this therapeutic strategy, the characteristics of treated patients, and care times in real-world clinical practice.

## Materials and methods

Study design and population

This was an observational, descriptive, retrospective study conducted at the Hospital de Especialidades, Centro Médico Nacional Siglo XXI, between March 2022 and May 2025. All consecutive patients with a clinical diagnosis of acute ischemic stroke who received intravenous thrombolysis and were cared for at our hospital during the study period were screened.

Inclusion and exclusion criteria

We included patients aged 18 years or older who were treated beyond the conventional 4.5-hour window. This included patients with a known symptom onset between 4.5 and 9 hours before treatment and patients with an unknown time of onset, including wake-up strokes. Patients with an unknown time of onset were considered for treatment when they were within nine hours of being found with focal neurological deficits.

We excluded patients who received thrombolysis within the conventional 4.5-hour window with a known time of onset, who had thrombolysis initiated at another institution prior to transfer, or whose medical records were incomplete and did not allow the study variables to be collected. We also excluded patients who received intravenous thrombolysis despite having any of the contraindications established in the 2019 American Heart Association/American Stroke Association guidelines for the early management of acute ischemic stroke [20].

Advanced imaging and patient selection

Patients evaluated with CT perfusion were considered eligible for thrombolytic treatment when a penumbra-to-core mismatch ratio >1.2 or an absolute penumbra-to-core volume difference >10 mL was identified, provided that the estimated ischemic core volume was <70 mL. The ischemic core was defined as cerebral blood flow <30% of normal, whereas critically hypoperfused tissue was defined by a time-to-maximum (Tmax) delay >6 seconds.

Patients selected using MRI were required to demonstrate a diffusion-weighted imaging (DWI)-FLAIR mismatch. This mismatch was defined as the presence of a hyperintense ischemic lesion on DWI that was not apparent on the corresponding FLAIR sequence.

All images obtained were manually interpreted, and the choice of advanced imaging modality was determined according to the clinical scenario and institutional availability. Apart from the time-based eligibility criteria, all other conventional eligibility criteria for intravenous thrombolysis were applied according to the clinical assessment and available diagnostic information.

Thrombolytic treatment

All patients received intravenous alteplase (recombinant tissue plasminogen activator, rt-PA) at a dose of 0.9 mg/kg (maximum 90 mg), with 10% of the total dose administered as an intravenous bolus over one minute and the remaining 90% as a continuous infusion over 60 minutes. No patient received tenecteplase.

Clinical assessment and data collection

For each included patient, demographic, clinical, temporal, vascular, and imaging variables were retrospectively collected from the medical records. Demographic variables included age and sex. Clinical variables included the affected vascular territory, affected artery, and baseline National Institutes of Health Stroke Scale (NIHSS) score. The NIHSS was used to quantify the severity of neurological impairment and was assessed according to the original scale described by Brott et al. [21].

Temporal variables included time from symptom onset to hospital arrival, time from hospital arrival to assessment by the stroke team, time from hospital arrival to completion of the imaging study, and time from hospital arrival to initiation of reperfusion treatment. The modality of advanced neuroimaging performed (CT perfusion or MRI) was also recorded.

Statistical analysis

All statistical analyses were performed using IBM SPSS Statistics for Windows, Version 27 (Released 2019; IBM Corp., Armonk, NY, USA). A descriptive analysis was performed for all study variables.

The distribution of quantitative variables was assessed using the Shapiro-Wilk test. Quantitative variables with a normal distribution were expressed as mean ± standard deviation (SD), whereas variables with a non-normal distribution were expressed as median and interquartile range (IQR). Qualitative variables were summarized using absolute frequencies (n) and percentages (%).

Ethical considerations

The study protocol was reviewed and approved by the Hospital de Especialidades, Centro Médico Nacional Siglo XXI local research committee under registration number R-2026-3601-014. The study was conducted in accordance with the applicable institutional and ethical standards for research involving human participants.

## Results

From March 2022 to May 2025,97 patients with acute ischemic stroke treated with intravenous thrombolysis were identified. Of these, 18 were excluded because of incomplete records or thrombolysis initiated at another institution. Of the remaining 79 patients, 14 (17.7%) were treated with thrombolysis outside the classic window or with an unknown onset time.

Of the 14 patients, 11 (79%) were female and three (21%) were male. The mean age was 67 ± 17.2 years; four (29%) were young patients, defined as those younger than 55 years. No strokes occurred in patients who were already hospitalized, and no patient died during hospitalization. The median NIHSS score was 9 (IQR, 6-10) points. Baseline characteristics are summarized in Table 1.

**Table 1:** Demographic, clinical, and treatment characteristics of patients treated with intravenous thrombolysis in the extended window or with unknown symptom onset (n=14) RMCA: right middle cerebral artery; LMCA: left middle cerebral artery; RVA: right vertebral artery; LVA: left vertebral artery; RPCA: right posterior cerebral artery; NIHSS: National Institutes of Health Stroke Scale; IQR: interquartile range; CT: computed tomography; MRI: magnetic resonance imaging

Parameters	
Age, mean ± SD	67 ± 17.2
Sex - n (%)	
Male	3 (21%)
Female	11 (79%)
Affected territory - n (%)	
Anterior circulation	11 (79%)
Posterior circulation	3 (21%)
Affected artery - n (%)	
LMCA	6 (43%)
RMCA	5 (36%)
RVA	1 (7%)
LVA	1 (7%)
RPCA	1 (7%)
NIHSS, median (IQR)	9 (6-10)
Symptom onset - n (%)	
Known	9 (64%)
Unknown	5 (36%)
Time intervals, median (IQR), min	
Onset-to-arrival	327 (280-420)
Door-to-stroke-team assessment	8 (5-22)
Door-to-imaging	30 (25-52)
Door-to-needle	74 (56-89)
Advanced imaging modality - n (%)	
CT perfusion	11 (79%)
MRI	3 (21%)

The most frequently affected territory was the anterior circulation in 11 (79%) patients. The affected arteries, in order of frequency, were the left middle cerebral artery (6, 43%); right middle cerebral artery (5, 36%); right vertebral artery (1, 7%); left vertebral artery (1, 7%); and right posterior cerebral artery (1, 7%).

Onset of symptoms was known in nine (64%) patients and unknown in five (36%) patients. The median time intervals were as follows: symptom onset to hospital arrival, 327 minutes (IQR, 280-420); door-to-stroke-team assessment, eight minutes (IQR, 5-22); door-to-imaging time, 30 minutes (IQR, 25-52); and door-to-needle time, 74 minutes (IQR, 56-89).

CT perfusion was performed in all nine (100%) patients with known symptom onset. Among patients with unknown onset, MRI was performed in three (60%) and CT perfusion in two (40%). Overall, CT perfusion was the most frequently used advanced imaging modality, performed in 11 (79%) patients, whereas MRI was performed in three (21%) (Figures 1-2).

**Figure 1:**
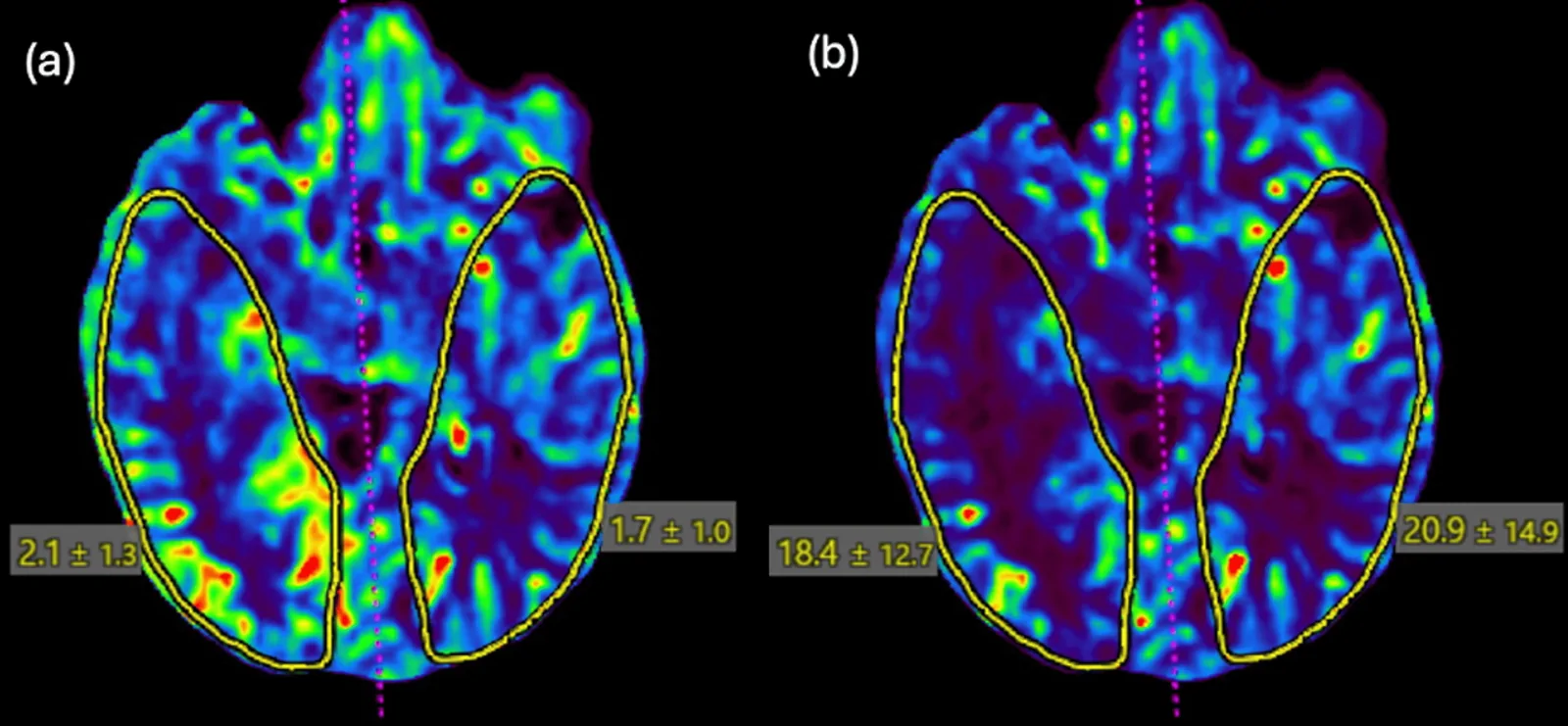
CT perfusion study CT perfusion study in a 73-year-old female patient with acute ischemic stroke of 7.5 hours' duration. The right temporo-occipital region shows preserved cerebral blood volume (a) and reduced cerebral blood flow (b), corresponding to an area of ischemic penumbra with potentially salvageable tissue.

**Figure 2:**
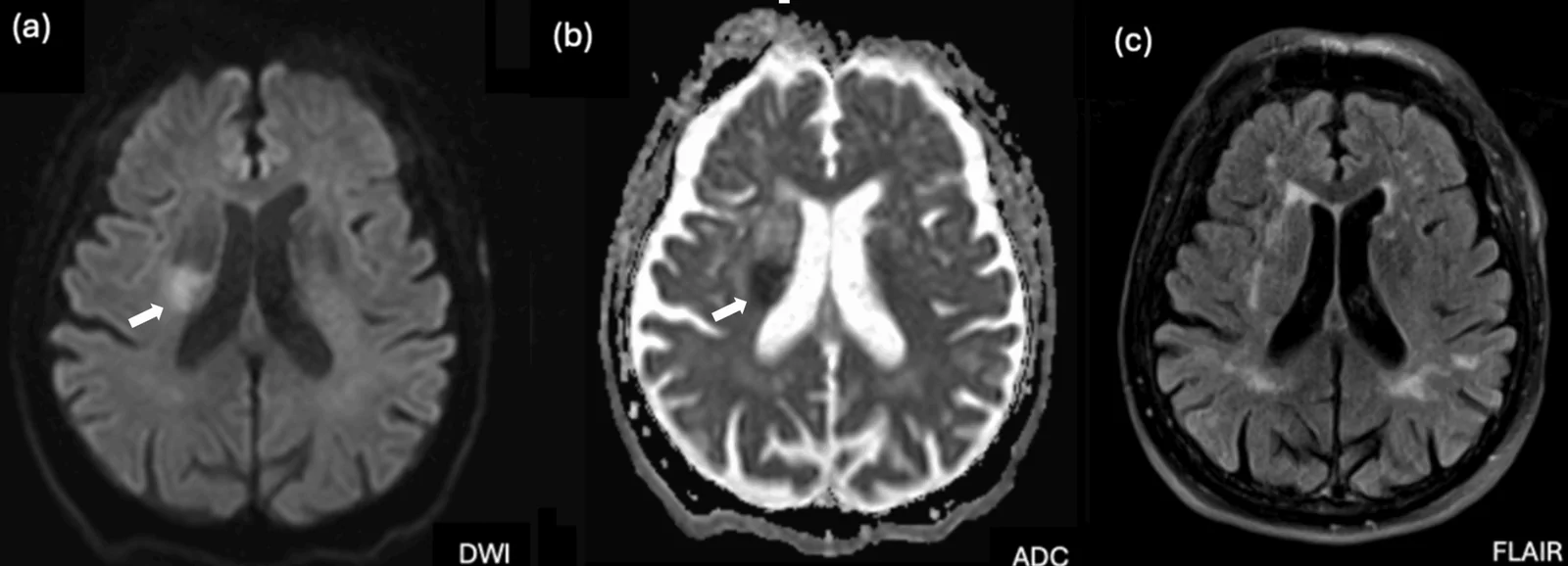
MRI study Brain MRI in a 64-year-old male patient with acute ischemic stroke of unknown onset, evaluated prior to intravenous thrombolysis. A hyperintense lesion in the right striatum (arrows) shows diffusion restriction on DWI (a) and ADC (b) sequences, without a corresponding lesion on the FLAIR sequence (c), consistent with a DWI-FLAIR mismatch and potentially salvageable tissue. DWI: diffusion-weighted imaging b=1000; ADC: apparent diffusion coefficient; FLAIR: fluid-attenuated inversion recovery

## Discussion

This study collected data on patients diagnosed with ischemic stroke who were evaluated and treated with thrombolysis between 4.5 and 9 hours after symptom onset, or with unknown onset, in order to analyze their characteristics, the care times at our institution, and the imaging studies performed to guide clinical decision-making.

In our cohort, 17.7% of patients with acute ischemic stroke received intravenous thrombolysis outside the classic window or with unknown onset time. This proportion reflects the growing relevance of extended-window thrombolysis, particularly considering that a large proportion of patients arrive at the hospital after the 4.5-hour window recommended by current guidelines [18]. Thrombolysis for stroke of unknown onset has been recommended by the American Heart Association since 2018, while thrombolysis in a 4.5-9-hour window has been recommended by the European Stroke Organisation since 2021, in both cases using mismatch criteria on CT or MRI. Both recommendations were based on the WAKE-UP and EXTEND trials, which demonstrated efficacy [19]. Mexico lacks an epidemiological registry of stroke patients, so data on extended-window thrombolysis remain scarce. A recent study conducted at a tertiary private hospital in Mexico City reported that approximately 15% of thrombolysed patients received treatment outside the classic window (>4.5 hours) [22], and this situation is expected to change with the new stroke care models that have been implemented across various institutions [23].

Our results showed a median NIHSS of 9, no in-hospital deaths, and a predominance of female patients, suggesting a clinical profile of moderate severity and a hospital course free of mortality. These findings are consistent with current evidence supporting the safety of thrombolysis beyond 4.5 hours in patients selected by neuroimaging. For example, the systematic review by Ahmad et al. demonstrated that intravenous thrombolysis in the extended window up to nine hours and in patients with unknown onset improves functional outcomes without increasing the risk of symptomatic intracranial hemorrhage (sICH) or death [24].

Similarly, the meta-analysis by Jia et al. found that, compared with standard treatment, thrombolysis beyond 4.5 hours increases the likelihood of functional independence at 90 days (mRS 0-2) without affecting mortality [18]. Our findings are in line with this: no in-hospital mortality was documented, which, although a limited outcome measure, is consistent with a low risk of adverse effects when patient selection is adequate.

Recent evidence from the HOPE trial is particularly relevant: in patients selected by cerebral perfusion imaging, the use of alteplase between 4.5 and 24 hours doubled the likelihood of functional independence compared with standard care, although with an increase in sICH and no difference in mortality [25]. This trial reinforces the notion that extended-window thrombolysis can be safe and effective in real-world clinical scenarios, similar to our experience.

Other systematic reviews confirm these findings. Palaiodimou et al. reported that extended-window thrombolysis is consistently associated with better functional outcomes without an increase in mortality, although at the expense of a higher risk of hemorrhage [19]. Ahmad et al. and Shi et al. also highlight that, as with mechanical thrombectomy in the late window, outcomes largely depend on appropriate patient selection using clinical and imaging criteria [24,26].

In our study, four (28%) of the patients treated in the extended window corresponded to cases of stroke in young patients (<55 years). This subgroup merits attention, as recent trials suggest that late-window thrombolysis may be particularly beneficial in patients with a lower comorbidity burden and greater expected functional recovery [27].

Taken together, our findings are consistent with the growing body of evidence on intravenous thrombolysis outside the classic window and support its feasibility when careful patient selection is applied. The absence of in-hospital mortality in our cohort reinforces the notion that clinical implementation of these protocols can improve access to reperfusion therapy in settings where thrombectomy is not always available and patients present late for medical care.

This study has several limitations. Its retrospective design and small sample size limit the generalizability of the findings and may introduce selection bias. Furthermore, the lack of long-term functional follow-up and of systematic recording of treatment-associated complications, such as symptomatic or asymptomatic intracranial hemorrhage, precluded the assessment of treatment safety and 90-day functional outcomes. In addition, other potential confounders were not assessed, particularly premorbid frailty, which has been independently associated with higher early mortality after ischemic stroke and with attenuated neurological improvement following intravenous thrombolysis [28]. Since frailty was not systematically documented in the medical records, it could not be evaluated, and the absence of in-hospital mortality in our cohort should be interpreted with caution.

## Conclusions

In this cohort, approximately one in every six thrombolysed patients received treatment within an extended therapeutic window or with an unknown onset time, reflecting the growing relevance of advanced neuroimaging-guided selection in clinical practice. Our findings suggest that implementation of extended-window thrombolysis protocols is feasible at referral centers in Mexico and may expand access to reperfusion therapies in patients who would traditionally be excluded by strictly time-based criteria. However, given the descriptive design, the absence of standardized monitoring of early complications such as symptomatic and asymptomatic intracranial hemorrhage, and the lack of adjustment for potential confounders such as premorbid frailty, these results should not be interpreted as evidence of safety or efficacy.

Larger, prospective, multicenter studies are needed to further evaluate the safety and effectiveness of advanced imaging-guided thrombolysis in the extended therapeutic window. Such studies should incorporate standardized monitoring of early complications, systematic assessment of premorbid frailty and other potential confounders, and long-term functional outcomes.

## References

[1] Feigin VL, Brainin M, Norrving B (2025). World Stroke Organization: Global Stroke Fact Sheet 2025. Int J Stroke.

[2] Saini V, Guada L, Yavagal DR (2021). Global epidemiology of stroke and access to acute ischemic stroke interventions. Neurology.

[3] Feigin VL, Stark BA, Johnson CO (2021). Global, regional, and national burden of stroke and its risk factors, 1990-2019: a systematic analysis for the Global Burden of Disease Study 2019. Lancet Neurol.

[4] Fajardo-Dolci G, Tapia-Conyer R, Lozano R (2023). The burden of disease in Mexico from 1990 to 2021. Gac Méd Méx.

[5] National Institute of Neurological Disorders and Stroke rt-PA Stroke Study Group (1995). Tissue plasminogen activator for acute ischemic stroke. N Engl J Med.

[6] Al-Ajlan FS, Alkhiri A, Alamri AF (2024). Golden hour intravenous thrombolysis for acute ischemic stroke: a systematic review and meta-analysis. Ann Neurol.

[7] Psychogios K, Tsivgoulis G (2022). Intravenous thrombolysis for acute ischemic stroke: why not?. Curr Opin Neurol.

[8] Bonifacio-Delgadillo DM, Castellanos-Pedroza E, Martínez-Guerra BA, Sánchez-Martínez CM, Marquez-Romero JM (2023). Delivering acute stroke care in a middle-income country. The Mexican model: "ResISSSTE Cerebro". Front Neurol.

[9] Demeestere J, Wouters A, Christensen S, Lemmens R, Lansberg MG (2020). Review of perfusion imaging in acute ischemic stroke: from time to tissue. Stroke.

[10] Garcia-Esperon C, Raposo N, Seners P, Spratt N, Parsons M, Olivot JM (2021). Role of neuroimaging before reperfusion therapy. Part 1 - IV thrombolysis - review. Rev Neurol (Paris).

[11] Thomalla G, Simonsen CZ, Boutitie F (2018). MRI-guided thrombolysis for stroke with unknown time of onset. N Engl J Med.

[12] Leira EC, Muir KW (2019). EXTEND trial. Stroke.

[13] Amiri H, Bluhmki E, Bendszus M (2016). European Cooperative Acute Stroke Study-4: extending the time for thrombolysis in emergency neurological deficits ECASS-4: ExTEND. Int J Stroke.

[14] Altersberger VL, Sibolt G, Enz LS (2023). Intravenous thrombolysis 4.5-9 hours after stroke onset: a cohort study from the TRISP collaboration. Ann Neurol.

[15] Günkan A, Ferreira MY, Vilardo M (2025). Thrombolysis for ischemic stroke beyond the 4.5-hour window: a meta-analysis of randomized clinical trials. Stroke.

[16] Imtiaz M, Zaman MA, Naseer A, Kalsoom S (2024). Role of tenecteplase in ischemic stroke after 4.5 hours: an evaluation of the TRACE-III trial. Expert Rev Clin Pharmacol.

[17] Simpkins AN, Tahsili-Fahadan P, Buchwald N (2021). Adapting clinical practice of thrombolysis for acute ischemic stroke beyond 4.5 hours: a review of the literature. J Stroke Cerebrovasc Dis.

[18] Jia X, Wang W, Wu B, Sun X (2021). Intravenous thrombolysis for acute ischemic stroke with extended time window. Chin Med J (Engl).

[19] Palaiodimou L, Papageorgiou NM, Safouris A (2025). Efficacy and safety of intravenous thrombolysis in the extended time window for acute ischemic stroke: a systematic review and meta-analysis. J Clin Med.

[20] Powers WJ, Rabinstein AA, Ackerson T (2019). Guidelines for the early management of patients with acute ischemic stroke: 2019 update to the 2018 guidelines for the early management of acute ischemic stroke: a guideline for healthcare professionals from the American Heart Association/American Stroke Association. Stroke.

[21] Brott T, Adams HP Jr, Olinger CP (1989). Measurements of acute cerebral infarction: a clinical examination scale. Stroke.

[22] Milpas Muñoz LD, Gutiérrez Romero A, Medina-Rioja R, Garcia-Santos RA, Gonzalez Chon O, Gasca Saldaña D (2025). Intravenous thrombolysis administration rate in patients with ischemic stroke at a tertiary private hospital in Mexico City. Cureus.

[23] Arauz A, Mendez B, Soriano-Navarro E (2019). Frequency of intravenous thrombolysis in Mexican patients with acute ischemic stroke. Int J Stroke.

[24] Ahmad M, Ranasinghe CA, Abu-Sa'da MO (2025). Efficacy and safety of intravenous thrombolysis beyond 4.5 hours in ischemic stroke: a systematic review and meta-analysis. Diagnostics (Basel).

[25] Zhou Y, He Y, Campbell BC (2025). Alteplase for acute ischemic stroke at 4.5 to 24 hours: the HOPE randomized clinical trial. JAMA.

[26] Shi Z, Zheng S, Jiang X, Wang X, Geng Y (2025). Late time window endovascular treatment for acute ischemic stroke: rethinking the role of simplified imaging. BMC Neurol.

[27] Galecio-Castillo M, Farooqui M, Hassan AE (2023). Clinical and safety outcomes of endovascular therapy 6 to 24 hours after large vessel occlusion ischemic stroke with tandem lesions. J Stroke.

[28] Evans NR, Wall J, To B, Wallis SJ, Romero-Ortuno R, Warburton EA (2020). Clinical frailty independently predicts early mortality after ischaemic stroke. Age Ageing.

